# Sleep Problems and Social Impairment in Psychosis: A Transdiagnostic Study Examining Multiple Social Domains

**DOI:** 10.3389/fpsyt.2020.00486

**Published:** 2020-05-28

**Authors:** Jack J. Blanchard, Christina L. G. Savage, Ryan D. Orth, Anyela M. Jacome, Melanie E. Bennett

**Affiliations:** ^1^Department of Psychology, University of Maryland, College Park, College Park, MD, United States; ^2^Department of Psychiatry, University of Maryland, Baltimore, Baltimore, MD, United States; ^3^Department of Psychiatry, Baltimore VA Medical Center, Baltimore, MD, United States

**Keywords:** psychotic disorders, sleep disturbance, social functioning, social relationships, transdiagnostic, clinical symptoms

## Abstract

Psychotic disorders are characterized by profound social impairment. An accumulation of research has explored the contribution of symptoms, cognitive functioning, and behavioral skills deficits to this social dysfunction. Recent research indicates that sleep disturbance has significant social implications in nonclinical populations—this research suggests that sleep problems may also be relevant to understanding social impairment in psychosis. This study adopted a symptom-oriented dimensional approach to examine how sleep disturbance and sleep-related impairment are related to multiple social domains within a transdiagnostic sample (N = 90). This sample included individuals with a variety of psychotic disorders (n = 75) along with healthy non-clinical participants (n = 15) to ensure sampling across the full range of sleep problems and social functioning. Social domains spanned self-reported perceptions of social relationships, social functioning in the community, and behavioral assessments of social competence. We hypothesized that greater sleep disturbance and sleep-related impairment would be associated with more negative or problematic perceptions of social relationships (i.e., less social support, less companionship, and greater distress), poorer social functioning in the community, smaller social networks, and poorer behavioral ratings of social competency. Results supported these hypotheses indicating that sleep disturbance and sleep-related impairment have widespread deleterious impacts on perceptions of social relationships, social functioning, and competence. Sleep disturbance retained associations with perceptions of social relationships, social functioning, and social competence even after controlling for total symptoms or cognitive functioning. These findings indicate that sleep problems may have important implications for fully understanding the causes of social impairment in psychosis.

## Introduction

Psychotic disorders, such as schizophrenia and bipolar disorder with psychosis, lead to profound and enduring social impairment (1–3). This impairment includes greater social isolation, lower rates of marriage, fewer friends, and strained family relationships (4, 5). When individuals with psychotic disorders do interact socially, they often have difficulty with the basic skills necessary to have a successful social interaction (6–8). This social impairment persists following stabilization of psychotic symptomatology with medication (9, 10). In a recent 20-year outcome study of individuals initially admitted with a first episode of psychosis, results indicated stable impairment in social functioning across psychotic diagnoses (11).

Social impairment in psychosis is determined by multiple factors. Findings in schizophrenia indicate that social impairment is a consequence of the combined effects of negative symptoms (12), cognitive impairment [e.g., (13, 14)], social cognitive impairment (15), and poor functional capacity or social skill (6–8). These factors have been shown to interact to result in social impairment in schizophrenia (16). A similar pattern of results has been obtained in bipolar disorder with negative symptoms, cognitive impairment, and poor functional capacity associated with poor community functioning (17).

Recent research suggests the novel idea that sleep may be an important consideration in understanding social impairment in psychosis. Sleep disturbances, such as insomnia, have been found to be related to a variety of mental disorders (18, 19). As a result, sleep disturbance has increasingly been viewed as a transdiagnostic factor contributing to psychiatric symptomatology and functional impairment (20, 21). Research indicates that sleep may be especially important in psychosis given that sleep disturbances and sleep disorders occur frequently in psychotic disorders [e.g., (22–24)] and are evident early in the course of psychosis (25, 26). Importantly, as reviewed below, sleep problems are tied to a variety of social difficulties.

Gordon et al. (27) have proposed a model outlining the bidirectional effects of sleep and social processes [also see review by Troxel (28) for a similar bidirectional perspective]. In this model, sleep has widespread impacts on social processes from social cognition to interpersonal behavior. Further, one’s social environment and experiences can have both positive and negative implications for various sleep parameters (27). Evidence supporting these bidirectional models comes from a variety of sources. Poor sleep quality, including even mild insomnia, has adverse impacts on overall quality of life including reports of poorer social functioning (29, 30). Marital relationship quality has been found to be related to sleep disturbances [see (28)] and perceived better relationship quality with relatives and friends has been found to be associated with better sleep quality (31). Supportive social relationships have been shown to be related to better sleep quality while aversive or strained social ties are related to poorer sleep quality (32–34). Finally, loneliness has been shown to be associated with poorer sleep efficiency (35) and sleep-related daytime dysfunction (36).

Beyond cross-sectional findings showing an association between social relationships and sleep, other methods have been used to directly test the causal role that sleep may have in social outcomes. In a 3-year longitudinal study, Tavernier and Willoughby (37) found bidirectional effects of sleep problems and poorer social ties with a mediating role of affect regulation. Using daily sleep diaries, Gordon and Chen (38) demonstrated that relatively poorer sleep leads to greater interpersonal conflict the following day (even after controlling for prior conflict). Providing social rejection feedback has been shown to lead to poorer subsequent sleep (27). Within couples’ interactions, poorer prior night sleep has been found to be related to a lower ratio of positive to negative affect, lower empathic accuracy, and less conflict resolution (38). Simon and Walker (39) have shown that experimental sleep deprivation can lead to social withdrawal and feelings of loneliness. Importantly, it was also demonstrated that when sleep deprived, individuals behave in ways that lead observers to perceive the individual as lonely and observers reported that they would be less willing to interact socially or collaboratively with the sleep deprived individual (39). Simon and Walker (39) concluded that sleep loss leads to a self-reinforcing cycle of social separation and withdrawal. Finally, sleep intervention for insomnia has been shown to improve self-reported social functioning [e.g., (40)], and treatments for obstructive sleep apnea have been shown to improve social functioning (41) and marital satisfaction (42).

Less is understood regarding the social implication of sleep in psychosis. In schizophrenia, poor sleep quality has been found to be associated with lower self-reported quality of life (43, 44) including less enjoyment and satisfaction with social relationships (45). Relationships between sleep quality and quality of life (including satisfaction with social relationships) have been shown to persist even after controlling for depression or medication side effects (45). Beyond ratings of satisfaction, lower sleep quality has been found to be related to poorer personal and social functioning in individuals with schizophrenia (46). Liu et al. (47) examined self-reported symptoms of obstructive sleep apnea (OSA; snoring, pauses in breathing during sleep, and disrupted sleep) within individuals with psychotic disorders and found that reports of pauses in breathing were associated with poorer overall health-related quality of life, including lower independent living and lower well-being (but not social relations). Findings from Liu et al. (47) are somewhat limited in that only single items were used to assess OSA-related symptoms and these symptoms were dichotomously scored as present or absent. In addition to research conducted on individuals with psychosis, other research indicates that sleep problems are related to poor role and social functioning in those at clinical high-risk for psychosis (48).

Consistent with the research in non-clinical populations, the existing literature suggests potential links between sleep disturbance and social impairment in psychosis. However, this sleep research in psychosis is limited in that few studies have examined objective indicators of social functioning [e.g., (46)] and most studies have examined social functioning at a very broad level focusing on quality of life or satisfaction with social functioning. Further, we are not aware of any study that has examined the impact of sleep on self-reports of social support (33, 34) or other perceptions of social relationships [e.g., loneliness (35)] that have been found to be associated with sleep quality. Finally, no study has examined how sleep is related to actual social behavior in psychotic disorders using assessments of social skill or competence—an important consideration given the clinical importance of skills deficits in determining functional impairment in psychosis (7, 8, 16, 17).

Guided by the NIMH research domain criteria (RDoC) framework (49–51), we adopted a symptom-oriented dimensional approach to examine how sleep disturbance and sleep-related impairment are related to multiple social domains within a transdiagnostic sample of individuals (N = 90). This sample included individuals with a variety of psychotic disorders (n = 75) along with healthy non-clinical participants (n = 15) to ensure sampling across the full range of sleep problems and social functioning. Social domains spanned self-reported perceptions of social relationships, functioning in the community, and behavioral assessments of social competence. We hypothesized that greater sleep disturbance and sleep-related impairment would be associated with more negative or problematic perceptions of social relationships (i.e., less social support, less companionship, and greater distress), poorer social functioning in the community, smaller social networks, and poorer behavioral ratings of social competency.

## Materials and Methods

### Participants

Participants were enrolled in a larger ongoing grant-funded project examining social affiliative deficits from an RDoC perspective (National Institutes of Health grant R01MH110462). The mixed sample (N = 90) included clinical (n = 75) and non-clinical community (n = 15) participants. Participants diagnosed with a psychotic disorder (e.g., schizophrenia/schizoaffective disorder, delusional disorder, major depression with psychosis) were recruited from outpatient mental health clinics in the Baltimore and Washington, D.C. metro areas. Demographic and diagnostic characteristics of the study sample are summarized in **Table 1**. Diagnoses were determined with the Structured Clinical Interview for DSM-5 (SCID-5) (52). The majority of clinical participants were prescribed various forms of antipsychotic medication, including atypical antipsychotics (n = 46, 61%), typical antipsychotics (n = 10; 13%), or a combination of atypical and typical antipsychotics (n = 10; 13%). Other prescribed medications for clinical participants included antidepressants (n = 42, 61%), antianxiety (n = 32, 52%), mood stabilizers (n = 30, 40%), and antiparkinsonian medications (n = 21, 28%). We have previously reported on symptom correlates of sleep problems in this sample (53).

**Table 1:** Sample characteristics (N = 90).

	Mean (SD) or n (percent)
Age (years)	44.44 (11.66)
Sex	
Male	55 (61.1%)
Female	35 (38.9%)
Race	
African-American	66 (73.3%)
White	19 (21.1%)
Asian	2 (2.2%)
More than one race	3 (3.3%)
Ethnicity	
Non-Hispanic or Latino	82 (91.1%)
Hispanic or Latino	7 (7.8%)
Unknown	1 (1.1%)
Marital Status	
Married	6 (6.7%)
Divorced/separated	13 (14.4%)
Never married/single	71 (78.9%)
Education (years)	12.78 (2.32)
Has a paying job	
Yes	29 (32.2%)
No	61 (67.8%)
Diagnosis	
Schizophrenia	34 (37.8%)
Schizoaffective Bipolar Type	13 (14.4%)
Schizoaffective Depressive Type	13 (14.4%)
Delusional disorder	1 (1.1%)
BP I w/ psychotic features	8 (8.9%)
MDD w/ psychotic features	6 (6.7%)
No diagnosis (healthy control)	15 (16.7%)

BP, Bipolar; MDD, Major Depressive Disorder.

Inclusion criteria for clinical participants included (1) aged 18–60, (2) lifetime history of a psychotic disorder, (3) clinical stability (i.e., no inpatient hospitalizations for 3 months before enrollment, no changes in psychoactive medication in the 4 weeks before enrollment) as indicated by approval of clinician and medical record review, and (4) fluent in English. Community participants were recruited *via* online advertisements, and inclusion criteria included (1) aged 18–60, (2) no current clinical disorder or psychiatric medications, (3) no lifetime history of a psychotic or mood disorder, (4) no avoidant, paranoid, schizotypal, or schizoid personality disorder, and (5) fluent in English. Exclusion criteria for all participants included (1) current substance use disorder, (2) neurological conditions (e.g., epilepsy, multiple sclerosis), (3) evidence of intellectual disability as determined by medical history or cognitive testing, (4) any history of serious head injury, (5) any MRI contraindications (e.g., MRI unsafe metal in body, weight that exceeds the limitations of MRI machine), and (6) unwillingness to be videotaped during study participation.

### Assessment of Sleep

Sleep assessments utilized the National Institutes of Health Patient-Reported Outcomes Measurement Information System (PROMIS™) Sleep Disturbance and Sleep-Related Impairment short-form scales (54). The Sleep Disturbance scale includes items such as “I had difficulty falling asleep” and “I had trouble staying asleep”. The Sleep-Related Impairment scale includes items such as “I had a hard time concentrating because of poor sleep” and “I had a hard time getting things done because I was sleepy”. These scales were developed using rigorous item-response theory methods as well as clinical judgement from content experts. The PROMIS™ Sleep Disturbance scale has demonstrated high convergent validity with the Pittsburgh Sleep Quality Index and both the PROMIS™ Sleep Disturbance and Sleep-Related Impairment scales are capable of differentiating healthy individuals from those with clinically diagnosed sleep disorders (54). The PROMIS™ sleep scales have also been shown to be sensitive to treatment effects of positive airway pressure therapy (55). Although not intended to measure symptoms of specific sleep disorders, the PROMIS™ scales do tap sleep quality and sleep dissatisfaction; thus, they are useful in assessing global severity of insomnia (54).

### Assessment of Functioning

The Adult Social Relationships Scales (ASRS) (56) consists of six self-report scales assessing for participant appraisals of social relationships over the past month across multiple domains. Domains assessed include perceived social rejection, perceived hostility, loneliness, friendship, instrumental support, and emotional support. The ASRS is part of the NIH Toolbox for the Assessment of Neurological and Behavioral Function and each scale has good internal reliability and concurrent validity with other instruments (56).

The Specific Levels of Functioning Scale (SLOF) (57) is a self-report measure that assesses community functioning. It consists of four subscales related to social and community functioning (Interpersonal Relationships, Social Acceptability, Involvement in Activities and Work Skills). The SLOF has been judged to be the best available measure of real-world functioning (21). Consistent with the procedures used in other samples with psychosis [see (21)], participants completed a 30-item self-report measure of their “*typical* level of functioning” rated on a five-point Likert scale. Higher ratings indicate better functioning.

The Social Network Index (SNI) (58) is a 13-item self-report measure assessing for social network size. Social network size is the total number of individuals whom the respondent has had contact with at least once in the previous two weeks. The SNI has been used to study social relationships in samples at ultra-high risk for psychosis (59) and has been shown to relate to neuromorphology and brain function in neuroimaging studies (60, 61).

The UCSD Performance-Based Skills Assessment Brief Version (UPSA-B) (7) utilizes the Financial and Communications subscales, two out of the five original subscales that make-up the full UPSA. The UPSA-B uses role-playing tasks to assess an individual’s capacity to perform everyday tasks as an indicator of their real-world functioning in the community. The financial skills subscale asks participants to count and make change using monetary props (i.e., US dollar bills and coins), and analyze and fill out a check for a utility bill. The Communication skills subscale asks participants to demonstrate their knowledge of using the telephone by rescheduling an appointment, dialing emergency services, and dialing directory assistance to obtain a telephone number. The total score ranges from 0 to 100 and is calculated by summing each of the two subscales with standardized scores ranging on a 0 to 50 scale. Higher scores indicate higher functional capacity. The UPSA-B has been found to be a valid behavioral measure of functional milestones and has good predictive validity for vocational outcomes (7). In a recent review (62), the UPSA was found to have robust relations with social functioning as assessed with the SLOF.

The MATRICS Consensus Cognitive Battery (MCCB) (63) is a cognitive battery designed specifically for people with psychosis. It includes 10 measures assessing for cognitive functioning in seven domains. Domains include speed of processing, attention, working memory, verbal learning, visual learning, reasoning and problem solving, and social cognition. Each of the measures included in the battery has demonstrated acceptable validity and test-retest reliability (63). The MCCB has been shown to be related to functional outcomes in schizophrenia [e.g., (64)].

### Assessment of Symptomology

Brief Psychiatric Rating Scale, expanded version (BPRS) (65), is a 24-item clinical interview designed to assess current clinical symptomatology as experienced over the previous week. For the purposes of this study, an overall BPRS score was used to assess total symptom severity. The BPRS has shown acceptable test-rest reliability, internal correlation coefficients, and discriminant validity (66).

### Procedures

Data were collected from within a larger fMRI study exploring the links between social affiliation and neurological threat response along with social reward processing. Study procedures were approved by the University of Maryland School of Medicine Institutional Review Board. Participants completed a standardized informed consent process with trained recruiters and signed an informed consent document. A brief questionnaire was administered by trained study staff to verify that participants were competent to provide consent and understood the consent document. After the consent process, participants completed in-person clinical interviews and self-report paper-and-pencil questionnaires related to diagnoses, cognitive functioning, social affiliation, and social functioning. Participants were also compensated for their participation.

### Data Analysis

Descriptive statistics for the sleep and social functioning measures are presented in **Table 2**. As reflected in the T-score values, PROMIS™ scales of sleep disturbance (range = 28.90–76.50) and sleep-related impairment (range = 30.00–76.90) represented a wide range, including values that were greater than two standard deviations above the population average (54). There were no significant differences between male and female participants in sleep disturbance (t = 1.88, p = .07) or sleep impairment (t = 0.52, p = .61). Age was not correlated with either sleep disturbance (r = .14, p = .20) or sleep impairment (r = −.04, p = .72). Given these null results, sex and age were not considered in the analyses. Descriptive statistics for total symptoms and MATRICS cognitive measures are presented in **Table 3**. Correlational analyses were conducted to assess the relation between sleep and social measures with additional partial correlation analyses conducted to control for symptomatology and cognitive impairment.

**Table 2:** Descriptive statistics for sleep and social functioning assessments.

	M (SD)
Sleep Disturbance (n = 90)	17.74 (7.73)
T-Score	46.00 (10.58)
Sleep-Related Impairment (n = 90)	17.02 (7.21)
T-Score	49.37 (10.64)
Adult Social Relationship Scales	
Emotional Support (n = 90)	32.28 (7.45)
Instrumental Support (n = 89)	26.42 (8.84)
Friendship (n = 88)	25.41 (7.92)
Loneliness (n = 89)	10.82 (5.64)
Perceived Rejection (n = 89)	15.01 (6.99)
Hostility (n = 88)	14.67 (6.82)
Social Functioning (SLOF)	
Interpersonal (n = 90)	27.78 (6.08)
Social Acceptability (n = 90)	27.58 (2.67)
Social Function (n = 90)	55.36 (7.48)
Activities (n = 89)	52.21 (6.33)
Work Skills (n = 89)	26.54 (3.81)
Comm Living Skills (n = 88)	83.31 (9.19)
Social Network Size (n = 90)	11.16 (7.37)
Social Competence (UPSA)	
Financial (n = 89)	41.09 (8.12)
Communication (n = 90)	37.00 (9.10)

SLOF, Specific Level of Function Scale; UPSA, University of California, San Diego (UCSD) Performance-Based Skills Assessment.

**Table 3:** Descriptive statistics for symptoms and cognitive functioning.

	M (SD)
BPRS Total Symptom Score (n = 90)	37.24 (9.81)
MATRICS Consensus Cognitive Battery:	
Trail Making Test, Part A (n = 90)	49.69 (29.30)
Symbol Coding (n = 89)	40.46 (14.81)
Category Fluency (n = 88)	18.60 (5.80)
Verbal Learning (n = 90)	19.81 (6.67)
Spatial Span (n = 89)	12.97 (3.66)
Letter-Number Span (n = 89)	10.73 (4.30)
Mazes (n = 87)	9.53 (6.64)
Visuospatial Memory (n = 86)	15.83 (8.76)
Managing Emotions (n = 88)	88.96 (14.70)
Continuous Performance Test (n = 87)	3.40 (16.14)

## Results

Correlations between sleep variables and social variables are presented in **Table 4**. Results from the Adult Social Relationship Scale indicated that greater sleep disturbance and sleep-related impairment were related to self-reports of lower perceived emotional support (but not instrumental support), lower ratings of friendship and greater loneliness (range of rs = −.25 to −.28, ps < 05). Greater sleep disturbance and sleep-related impairment were related to greater social distress as reflected by associations with perceived social rejection and hostility from others (range of rs = .36 to .44, ps < .01).

**Table 4:** Social correlates of sleep disturbance and sleep-related impairment.

	Sleep Disturbance	Sleep-Related Impairment
**Adult Social Relationship Scales**		
Emotional Support	−.28**	−.25*
Instrumental Support	−.09	−.16
Friendship	−.27*	−.26*
Loneliness	.36**	.36**
Perceived Rejection	.36**	.41***
Hostility	.37***	.44***
**Social Functioning (SLOF)**		
Interpersonal	−.33***	−.36***
Social Acceptability	−.40***	−.50***
Overall Social Function	−.41***	−.47***
Activities	−.16	−.06
Work Skills	−.41***	−.49***
Community Living Skills	−.30**	−.25*
**Social Network**	−.22*	−.15
**Social Competence (UPSA)**		
Financial	−.17	−.12
Communication	−.26*	−.33***

SLOF, Specific Level of Function Scale; UPSA, University of California, San Diego (UCSD) Performance-Based Skills Assessment. *p < .05; **p < .01; ***p < .005.

With regard to social functioning, greater sleep disturbance and sleep-related impairment were associated with poorer functioning across all domains with the exception of activities (range of rs = −.25 to −.50, ps < .05). Turning to social network size, greater sleep disturbance (but not sleep-related impairment) was related to smaller social networks (r *=* −.22, p < .05). Finally, greater sleep disturbance and sleep-related impairment were related to poorer social competence as measured by ratings of communication skills (rs = −.26 and −.33, respectively, ps < .05) but not financial skills on the UPSA.

Given that symptoms and cognitive impairment can contribute to social dysfunction, we sought to determine if sleep disturbance continued to hold a relation with social variables after controlling for total symptoms on the BPRS (due to missing data, partial correlations range of n = 83–87) and after simultaneously controlling for all seven MATRICS subtests (due to missing data, partial correlations range of n = 72–76). Results of partial correlations are presented in **Table 5**. After controlling for the BPRS total symptom score, greater sleep disturbance continued to be related to more social distress as reflected by greater perceived rejection and hostility. Sleep disturbance also remained associated with a range of social functioning measures except activities and community living skills. Finally, greater sleep disturbance remained associated with poorer social competence as assessed by the UPSA communication scale. A similar pattern of results was obtained after controlling for cognitive performance with sleep disturbance retaining associations across perceptions of social relationships, social functioning as well as social competence (communication).

**Table 5:** Social correlates of sleep disturbance controlling for symptoms and cognitive impairment.

	Sleep Disturbance	
	Controlling for Symptoms partial-r	Controlling for Cognitive Impairment partial-r
**Adult Social Relationship Scales**		
Emotional Support	−.18	−.19
Instrumental Support	−.08	−.14
Friendship	−.17	−.18
Loneliness	.17	.34**
Perceived Rejection	.27*	.33***
Hostility	.30**	.43***
**Social Functioning (SLOF)**		
Interpersonal	−.23*	−.28*
Social Acceptability	−.34***	−.46***
Overall Social Function	−.32***	−.41***
Activities	.06	−.04
Work Skills	−.38***	−.39***
Community Living Skills	−.11	−.20
**Social Network**	−.11	−.04
**Social Competence (UPSA)**		
Financial	−.07	−.15
Communication	−.22*	−.25*

SLOF, Specific Level of Function Scale; UPSA, University of California, San Diego (UCSD) Performance-Based Skills Assessment. *p < .05; **p < .01; ***p < .005.

## Discussion

This study sought to examine the contribution of sleep disturbance and sleep-related impairment to a variety of social domains within a transdiagnostic sample including psychotic disorders. Sleep disturbance and sleep-related impairment were assessed with standardized and validated measures. A strength of the study was that social assessments spanned several domains including self-reported perceptions of social relationships, social functioning in the community, social network size, and behavioral assessments of social competence.

Regarding perceptions of social relationships, sleep disturbance and sleep-related impairment were related to reports of lower emotional (but not instrumental) support as well as lower reports of friendship and greater loneliness. The results are consistent with findings from non-clinical samples indicating that lower social support and greater loneliness are related to lower sleep quality (32–35) and greater sleep-related impairment (36) and extend these findings to samples with psychosis. Further, both facets of sleep problems were related to greater perceptions of social rejection and hostility from others. These results are consistent with prior findings that social rejection is related to sleep disturbance in healthy individuals (27). The results for perceptions of social relationships as hostile are potentially consistent with findings that greater paranoid ideation is related to poorer sleep quality in clinical [e.g., (67)] and non-clinical [e.g., (68)] samples. The association between greater emotional support and friendship and lower sleep disturbance found in zero-order correlations were no longer maintained when controlling for symptoms or cognitive functioning. However, greater perceived rejection and hostility remained associated with greater sleep disturbance even after controlling for total symptoms and overall cognitive functioning (and loneliness remained related to sleep disturbance after controlling for cognitive performance). This pattern of results indicates that sleep disturbance may have an especially robust impact on negative perceptions of social relations potentially leading to a sense of rejection and perceptions of being treated in a hostile manner by others.

Greater sleep disturbance and sleep-related impairment were associated with social dysfunction across a variety of domains including poorer overall functioning and poorer work functioning. Notably, these correlates reflected medium to large effect sizes. Importantly, these associations between sleep disturbance and poorer social functioning persisted even after controlling for symptoms or cognitive functioning (factors known to contribute to social impairment). These results suggest that sleep disturbance may have a unique contribution to social impairment in psychosis above and beyond symptomatology and cognitive impairment. Our findings replicate and extend those of Afonso et al. (46) indicating that lower sleep quality was related to poorer personal and social functioning in individuals with schizophrenia.

Findings indicated that sleep disturbance and sleep-related impairment were related to behavioral ratings of social competence in the domain of communication (but not finance). Sleep disturbance remained associated with social competency ratings in communication even after controlling for symptoms and cognitive impairment. This is the first demonstration that we are aware of showing that sleep disturbance manifests in behavior with impaired social skill. Given the contribution of social competence to functional impairment (7, 8, 16), this suggests that sleep disturbance may be a relevant factor to consider in models that seek to understand skills deficits and functional impairment in psychotic disorders.

Overall, our results indicate that sleep problems in psychosis are associated with a variety of social problems involving negative social perceptions, poor functioning in the community, and diminished social skill. These results fit with prior work in non-clinical populations indicating the important connections between sleep and social processes (27, 28). The current findings are cross-sectional; thus, we are not able to disentangle to what extent sleep problems precede and give rise to social problems [e.g., (38, 39)] or whether social difficulties contribute causally to sleep problems [e.g., (27)]. However, current models emphasize a bidirectional relationship between sleep and social behavior (27).

The present findings suggest several paths for future research. An examination of how sleep problems impact social functioning is needed to better understand the mechanisms underlying this association. Prior research suggests that sleep insufficiency may lead to behavioral alterations including a lower ratio of positive to negative affect, lower empathic accuracy, and less conflict resolution (38). Sleep loss may also contribute to feelings of loneliness and self-initiated social withdrawal that adversely impacts observers desire to interact with an individual (39).

Beyond clinical, self-report and behavioral assessments, future research should explore neural mechanisms that might underlie the connection between sleep and social dysfunction in psychosis. Simon and Walker (39) found that sleep deprivation results in social withdrawal based on reduced activity in theory of mind neural networks and increased activity in brain regions involved in perceiving threatening approach. Insomnia has been shown to increase amygdala activation and reduce prefrontal-amygdala connectivity (69–71). Given the role of the amygdala and related structures in negative affect and threat perception [e.g., (72, 73)], amygdala activation associated with sleep insufficiency may in part contribute to perceptions of social rejection and hostility. It is interesting that, paralleling findings of amygdala activation in schizophrenia (74), sleep deprivation has been found to result in elevated amygdala activity to both aversive and neutral stimuli (75). Thus, sleep insufficiency may lead to neural changes that contribute to the misinterpretation of neutral or ambiguous stimuli as threatening. Additional research is required to further examine if sleep insufficiency contributes to social impairment through changes in brain circuitry that are involved in social avoidance, threat perception, and negative affect.

Finally, the current results may have implications for developing interventions to improve social functioning in psychosis. Our findings suggest that sleep disturbance may contribute to social impairment beyond overall symptoms and cognitive impairment. Should this finding be replicated, such an association might indicate that targeting sleep disturbance could enhance functional outcomes in psychotic disorders. Prior research has indicated that sleep interventions can positively impact social functioning in non-psychiatric individuals [e.g., (40–42)]. Given the association between negative symptoms and social impairment in psychosis (12) and the relationship between sleep problems and negative symptoms (53), it is interesting to speculate that adding sleep interventions to treatments for negative symptoms and social impairment could potentiate clinical outcomes to further enhance recovery.

Several limitations constrain interpretation of the present findings. First, as noted previously, this is a cross-sectional study and we are not able to establish if sleep disturbance and related impairment are contributing to social problems or if social difficulties give rise to sleep problems. Second, while we adopted an RDoC approach (49–51) to examine transdiagnostic associations from a dimensional perspective, we were not able to explore the potential differential relations of sleep domains across different diagnoses or differences between clinical and non-clinical participants. Third, the majority of clinical participants were receiving various forms of medication and we are not able to examine how different types or dosages of medication may contribute to sleep problems. Finally, assessment of sleep disturbance and sleep-related impairment were limited to self-reports. We do not have information relevant to the presence of clinically diagnosed sleep disorders nor do we have information based on other methods such as sleep diaries or actigraphy—methods that may provide additional information on sleep parameters not captured in our current assessments.

In summary, consistent with our hypotheses, results indicated that sleep disturbance and sleep-related impairment have widespread deleterious impacts on perceptions of social relationships, social functioning, and social competence or behavioral skill. Sleep disturbance retained its associations with perceptions of social relationships, social functioning, and social competence even after controlling for total symptoms or cognitive functioning. These findings have implications for understanding the profound social impairment that occurs in psychosis and suggest novel approaches to treating social dysfunction in these disorders.

## Data Availability Statement

The datasets generated for this study can be found in the NIMH Data Archive (NDA study account in process).

## Ethics Statement

The studies involving human participants were reviewed and approved by Institutional Review Board, University of Maryland, Baltimore. The patients/participants provided their written informed consent to participate in this study.

## Author Contributions

JB and MB developed the study design. RO, AJ, and CS assisted with data collection. JB analyzed the data. All authors contributed to writing the manuscript.

## Funding

This work was supported by the National Institutes of Health grant (R01MH110462).

## Conflict of Interest

The authors declare that the research was conducted in the absence of any commercial or financial relationships that could be construed as a potential conflict of interest.

## References

[1] Simonsen C Sundet K Vaskinn A Ueland T Romm KL Hellvin T Psychosocial function in schizophrenia and bipolar disorder: relationship to neurocognition and clinical symptoms. J Int Neuropsychol Soc Soc (2010) 16:771–83. 10.1017/S1355617710000573 20509984

[2] van Rheenen TE Rossell SL Objective and subjective psychosocial functioning in bipolar disorder: an investigation of the relative importance of neurocognition, social cognition and emotion regulation. J Affect Disord (2014) 162:134–41. 10.1016/j.jad.2014.03.043 24767018

[3] Wiersma D Wanderling J Dragomirecka E Ganev K Harrison G an der Heiden WA Social disability in schizophrenia: its development and prediction over 15 years in incidence cohorts in six European centres. Psychol Med Med (2000) 30:1155–67. 10.1017/S0033291799002627 12027051

[4] Green MF Horan WP Lee J McCleery A Reddy LF Wynn JK Social disconnection in schizophrenia and the general community. Schizophr Bull (2018) 44:242–9. 10.1093/schbul/sbx082 PMC5814840 28637195

[5] Gayer-Anderson C Morgan C Social networks, support and early psychosis: a systematic review. Epidemiol Psychiatr Sci (2013) 22:131–46. 10.1017/S2045796012000406 PMC6998121 22831843

[6] Blanchard JJ Park SG Catalano LT Bennett ME Social affiliation and negative symptoms in schizophrenia: examining the role of behavioral skills and subjective responding. Schizophr Res (2015) 168:491–7. 10.1016/j.schres.2015.07.019 PMC4762010 26235753

[7] Mausbach BT Harvey PD Pulver AE Depp CA Wolyniec PS Thornquist MH Relationship of the Brief UCSD Performance-based Skills Assessment (UPSA-B) to multiple indicators of functioning in people with schizophrenia and bipolar disorder. Bipolar Disord (2010) 12:45–55. 10.1111/j.1399-5618.2009.00787.x 20148866 PMC2846793

[8] Mueser KT Bellack AS Douglas MS Morrison RL Prevalence and stability of social skill deficits in schizophrenia. Schizophr Res (1991) 5:167–76. 10.1016/0920-9964(91)90044-R 1931809

[9] Bellack AS Schooler NR Marder SR Kane JM Brown CH Yang Y Do clozapine and risperidone affect social competence and problem solving? Am J Psychiatry (2004) 161:364–7. 10.1176/appi.ajp.161.2.364 14754789

[10] Huxley N Baldessarini RJ Disability and its treatment in bipolar disorder patients. Bipolar Disord (2007) 9:183–96. 10.1111/j.1399-5618.2007.00430.x 17391360

[11] Velthorst E Fett AKJ Reichenberg A Perlman G van Os J Bromet EJ The 20-year longitudinal trajectories of social functioning in individuals with psychotic disorders. Am J Psychiatry (2016) 174:1075–85. 10.1176/appi.ajp.2016.15111419 PMC5474222 27978770

[12] Blanchard JJ Bradshaw KR Garcia CP Nasrallah HA Harvey PD Casey D Examining the reliability and validity of the Clinical Assessment Interview for Negative Symptoms within the Management of Schizophrenia in Clinical Practice (MOSAIC) multisite national study. Schizophr Res (2017) 185:137–43. 10.1016/j.schres.2017.01.011 28087270

[13] Cohen A Forbes CB Mann MC Blanchard JJ Specific cognitive deficits and differential domains of social functioning impairment in schizophrenia. Schizophr Res (2006) 81:227–38. 10.1016/j.schres.2005.09.007 16260120

[14] Jabben N Arts B van Os J Krabbendam L Neurocognitive functioning as intermediary phenotype and predictor of psychosocial functioning across the psychosis continuum: studies in schizophrenia and bipolar disorder. J Clin Psychiatry (2010) 71:764–74. 10.4088/JCP.08m04837yel 20122370

[15] Fett A-KJ Viechtbauer W Dominguez M-G Penn DL van Os J Krabbendam L The relationship between neurocognition and social cognition with functional outcomes in schizophrenia: a meta-analysis. Neurosci Biobehav Rev (2011) 35:573–88. 10.1016/j.neubiorev.2010.07.001 20620163

[16] Bowie CR Reichenberg A Patterson TL Heaton RK Harvey PD Determinants of real-world functional performance in schizophrenia subjects: correlations with cognition, functional capacity, and symptoms. Am J Psychiatry (2006) 163:418–25. 10.1176/appi.ajp.163.3.418 16513862

[17] Bowie CR Depp C McGrath JA Wolyniec P Mausbach BT Thornquist MH Prediction of real-world functional disability in chronic mental disorders: a comparison of schizophrenia and bipolar disorder. Am J Psychiatry (2010) 167:1116–24. 10.1176/appi.ajp.2010.09101406 PMC3694770 20478878

[18] Benca RM Obermeyer WH Thisted RA Gillin JC Sleep and psychiatric disorders: a meta-analysis. Arch Gen Psychiatry (1992) 49:651–68. 10.1001/archpsyc.1992.01820080059010 1386215

[19] Klingaman EA Brownlow JA Boland EM Mosti C Gehrman PR Prevalence, predictors and correlates of insomnia in US army soldiers. J Sleep Res (2017) 27:1–13. 10.1111/jsr.12612 PMC5895531 29024363

[20] Harvey AG Sleep and circadian rhythms in bipolar disorder: seeking synchrony, harmony, and regulation. Am J Psychiatry (2008) 7:820–9. 10.1176/appi.ajp.2008.08010098 18519522

[21] Harvey AG Murray G Chandler RA Soehner A Sleep disturbance as transdiagnostic: consideration of neurobiological mechanisms. Clin Psychol Rev (2011) 31:225–35. 10.1016/j.cpr.2010.04.003 PMC2954256 20471738

[22] Kaskie RE Graziano B Ferrarelli F Schizophrenia and sleep disorders: links, risks, and management challenges. Nat Sci Sleep (2017) 9:227–39. 10.2147/NSS.S121076 PMC5614792 29033618

[23] Klingaman EA Palmer-Bacon J Bennett ME Rowland LM Sleep disorders among people with schizophrenia: emerging research. Curr Psychiatry Rep (2015) 17:616. 10.1007/s11920-015-06167 26279058

[24] Wee ZY Yong SWL Chew QH Guan C Lee TS Sim K Actigraphy studies and clinical and biobehavioural correlates in schizophrenia: a systematic review. J Neural Transm (2019) 126:531–58. 10.1007/s00702-019-01993-2 30888511

[25] Davies G Haddock G Yung AR Mulligan LD Kyle SD A systematic review of the nature and correlates of sleep disturbance in early psychosis. Sleep Med Rev (2017) 31:25–38. 10.1016/j.smrv.2016.01.001 26920092

[26] Reeve S Nickless A Sheaves B Hodgekins J Stewart SLK Gumley A Sleep duration and psychotic experiences in patients at risk of psychosis: a secondary analysis of the EDIE-2 trial. Schizophr Res (2019) 204:326–33. 10.1016/j.schres.2018.08.006 PMC6406020 30121185

[27] Gordon AM Mendes WB Prather AA The social side of sleep: elucidating the links between sleep and social processes. Curr Dir Psychol Sci (2017) 26:470–5. 10.1177/0963721417712269 PMC5791747 29398789

[28] Troxel WM Robles TF Hall M Buysse DJ Marital quality and the marital bed: examining the covariation between relationship quality and sleep. Sleep Med Rev Rev (2007) 11:389–404. 10.1016/j.smrv.2007.05.002 PMC2644899 17854738

[29] Lallukka T Sivertsen B Kronholm E Bin YS Øverland S Glozier N Association of sleep duration and sleep quality with the physical, social, and emotional functioning among Australian adults. Sleep Health (2018) 4:194–200. 10.1016/j.sleh.2017.11.006 29555134

[30] Léger D Scheuermaier K Philip P Paillard M Guilleminault C SF-36: evaluation of quality of life in severe and mild insomniacs compared with good sleepers. Psychosom Med (2001) 63:49–55. 10.1097/00006842-200101000-00006 11211064

[31] Yao KW Yu S Cheng SP Chen IJ Relationships between personal, depression and social network factors and sleep quality in community-dwelling older adults. J Nurs Res (2008) 16:131–9. 10.1097/01.JNR.0000387298.37419.ff 18528819

[32] Ailshire JA Burgard SA Family relationships and troubled sleep among US adults: examining the influences of contact frequency and relationship quality. J Health Soc Behav (2012) 53:248–62. 10.1177/0022146512446642 PMC3674886 22653715

[33] Chung J Social support, social strain, sleep quality, and actigraphic sleep characteristics: evidence from a national survey of US adults. Sleep Health (2017) 3:22–7. 10.1001/jama.1997.03540480040036 28346146

[34] Kent RG Uchino BN Cribbet MR Bowen K Smith TW Social relationships and sleep quality. Ann Behav Med (2015) 49:912–7. 10.1007/s12160-015-9711-6 PMC4636437 25976874

[35] Cacioppo JT Hawkley LC Berntson GG Ernst JM Gibbs AC Stickgold R Do lonely days invade the nights? Potential social modulation of sleep efficiency. Psychol Sci (2002) 13:384–7. 10.1111/1467-9280.00469 12137144

[36] Hawkley LC Preacher KJ Cacioppo JT Loneliness impairs daytime functioning but not sleep duration. Health Psychol (2010) 29:124–9. 10.1037/a0018646 PMC2841303 20230084

[37] Tavernier R Willoughby T A longitudinal examination of the bidirectional association between sleep problems and social ties at university: the mediating role of emotion regulation. J Youth Adolesc (2015) 44:317–30. 10.1007/s10964-014-0107-x 24578222

[38] Gordon AM Chen S The role of sleep in interpersonal conflict: do sleepless nights mean worse fights? Soc Psychol Pers Sci (2014) 5:168–75. 10.1177/1948550613488952

[39] Simon EB Walker MP Sleep loss causes social withdrawal and loneliness. Nat Commun (2018) 9:3146. 10.1038/s41467-018-05377-0 30108218 PMC6092357

[40] Freeman D Sheaves B Goodwin GM Yu L-M Nickless A Harrison PJ The effects of improving sleep on mental health (OASIS): a randomised controlled trial with mediation analysis. Lancet Psychiatry (2017) 4:749–58. 10.1016/S2215-0366(17)30328-0 PMC5614772 28888927

[41] Johal A Battagel J Hector M Controlled, prospective trial of psychosocial function before and after mandibular advancement splint therapy. Am J Orthod Dentofacial Orthop (2011) 139:581–7. 10.1016/j.ajodo.2009.06.035 21536199

[42] McFadyen TA Espie CA McArdle N Douglas NJ Engleman HM Controlled, prospective trial of psychosocial function before and after continuous positive airway pressure therapy. Eur Respir J (2001) 18:996–1002. 10.1183/09031936.01.00209301 11829108

[43] Brissos S Afonso P Cañas F Bobes J Bernardo-Fernandez I Guzman C Satisfaction with life of schizophrenia outpatients and their caregivers: differences between patients with and without self-reported sleep complaints. Schizophr Res Treat (2013) 2013:502172. 10.1155/2013/502172 PMC3826335 24288609

[44] Hofstetter JR Lysaker PH Mayeda AR Quality of sleep in patients with schizophrenia is associated with quality of life and coping. BMC Psychiatry (2005) 5:13. 10.1186/1471-244X-5-13 15743538 PMC554780

[45] Ritsner M Kurs R Ponizovsky A Hadjez J Perceived quality of life in schizophrenia: relationships to sleep quality. Qual Life Res (2004) 13:783–91. 10.1023/B:QURE.0000021687.18783.d6 15129888

[46] Afonso P Brissos S Bobes J Cañas F Bernardo-Fernandez I Personal and social functioning and satisfaction with life in schizophrenia outpatients with and without sleep disturbances. Rev Port Psiquiatr Saúde Ment (2015) 1:33–40.

[47] Liu D Myles H Foley DL Watts GF Morgan VA Castle D Risk factors for obstructive sleep apnea are prevalent in people with psychosis and correlate with impaired social functioning and poor physical health. Front Psychiatry (2016) 7:139. 10.3389/fpsyt.2016.00139 27630581 PMC5005426

[48] Poe SL Brucato G Bruno N Arndt LY Ben-David S Gill KE Sleep disturbances in individuals at clinical high risk for psychosis. Psychiatry Res (2017) 249:240–3. 10.1016/j.psychres.2016.12.029 PMC5893278 28126579

[49] Cuthbert BN The RDoC framework: facilitating transition from ICD/DSM to dimensional approaches that integrate neuroscience and psychopathology. World Psychiatry (2014) 13:28–35. 10.1002/wps.20087 24497240 PMC3918011

[50] Insel TR The NIMH Research Domain Criteria (RDoC) project: precision medicine for psychiatry. Am J Psychiatry (2014) 171:395–7. 10.1176/appi.ajp.2014.14020138 24687194

[51] Insel T Cuthbert B Garvey M Heinssen R Pine DS Quinn K Research Domain Criteria (RDoC): toward a new classification framework for research on mental disorders. Am J Psychiatry (2010) 167:748–51. 10.1176/appi.ajp.2010.09091379 20595427

[52] First MB Williams JBW Karg RS Spitzer RL Structured Clinical Interview for DSM-5—research version (SCID-5 for DSM-5, research version; SCID-5-RV). Arlington, VA: American Psychiatric Association (2015).

[53] Blanchard JJ Andrea A Orth RD Savage C Bennett ME Sleep disturbance and sleep-related impairment are related to both positive and negative symptoms. Psychiatry Res (2020) 286:112857. 10.1016/j.psychres.2020.112857 32087449 PMC7416463

[54] Yu L Buysse DJ Germain A Moul DE Stover A Dodds NE Development of short forms from the PROMIS™ sleep disturbance and sleep-related impairment item banks. Behav Sleep Med (2012) 10:6–24. 10.1080/15402002.2012.636266 PMC3261577 22250775

[55] Donovan LM Rueschman M Weng J Basu N Dudley KA Bakker JP The effectiveness of an obstructive sleep apnea screening and treatment program in patients with type 2 diabetes. Diabetes Res Clin Pract (2017) 134:145–52. 10.1016/j.diabres.2017.10.013 PMC5724386 29054482

[56] Cyranowski JM Zill N Bode R Butt Z Kelly MA Pilkonis PA Assessing social support, companionship, and distress: National Institute of Health (NIH) Toolbox Adult Social Relationship Scales. Health Psychol (2013) 32:293–301. 10.1037/a0028586 23437856 PMC3759525

[57] Schneider LC Struening EL SLOF: a behavioral rating scale for assessing the mentally ill. Soc Work Res Abstr (1983) 19:9–21. 10.1093/swra/19.3.9 10264257

[58] Cohen S Doyle WJ Skoner DP Rabin BS Gwaltney JM Social ties and susceptibility to the common cold. JAMA (1997) 277:1940–4. 10.1001/jama.1997.03540480040036 9200634

[59] Robustelli BL Newberry RE Whisman MA Mittal VA Social relationships in young adults at ultra high risk for psychosis. Psychiatry Res (2017) 247:345–51. 10.1016/j.psychres.2016.12.008 PMC5217827 27987484

[60] Bickart KC Wright CI Dautoff RJ Dickerson BC Barrett LF Amygdala volume and social network size in humans. Nat Neurosci (2011) 14:163–4. 10.1038/nn.2724 PMC3079404 21186358

[61] Bickart KC Hollenbeck MC Barrett LF Dickerson BC Intrinsic amygdala–cortical functional connectivity predicts social network size in humans. J Neurosci (2012) 32:14729–41. 10.1523/JNEUROSCI.1599-12.2012 PMC3569519 23077058

[62] Szabo S Merikle E Lozano-Ortega G Powell L Macek T Cline S Assessing the relationship between performance on the University of California Performance Skills Assessment (UPSA) and outcomes in schizophrenia: a systematic review and evidence synthesis. Schizophr Res Treat (2018) 2018:9075174. 10.1155/2018/9075174 PMC6327277 30687553

[63] Nuechterlein KH Green MF Kern RS Baade LE Barch DM Cohen JD The MATRICS Consensus Cognitive Battery, part 1: test selection, reliability, and validity. Am J Psychiatry (2008) 165:203–13. 10.1176/appi.ajp.2007.07010042 18172019

[64] Shamsi S Lau A Lencz T Burdick KE DeRosse P Brenner R Cognitive and symptomatic predictors of functional disability in schizophrenia. Schizophr Res (2011) 126:257–64. 10.1016/j.schres.2010.08.007 PMC3050077 20828991

[65] Ventura J Lukoff D Nuechterlein K Liberman RP Green MF Shaner A Brief Psychiatric Rating Scale (BPRS) Expanded version (4.0) scales, anchor points and administration manual. Int J Methods Psychiatr Res (1993), 227–44.

[66] Kopelowicz A Ventura J Liberman RP Mintz J Consistency of Brief Psychiatric Rating Scale factor structure across a broad spectrum of schizophrenia patients. Psychopathology (2007) 41:77–84. 10.1159/000111551 18033976

[67] Mulligan LD Haddock G Emsley R Neil ST Kyle SD High resolution examination of the role of sleep disturbance in predicting functioning and psychotic symptoms in schizophrenia: A novel experience sampling study. J Abnorm Psychol (2016) 125:788–97. 10.1037/abn0000180 27362488

[68] Freeman D Stahl D McManus S Meltzer H Brugha T Wiles N Insomnia, worry, anxiety and depression as predictors of the occurrence and persistence of paranoid thinking. Soc Psychiatry Psychiatr Epidemiol (2012) 47:1195–203. 10.1007/s00127-011-0433-1 21928153

[69] Krause A Simon EB Mander BA Greer SM Saletin JM Goldstein-Piekarski AN The sleep-deprived human brain. Nat Rev Neurosci (2017) 18:404–18. 10.1038/nrn.2017.55 PMC6143346 28515433

[70] Motomura Y Kitamura S Oba K Terasawa Y Enomoto M Katayose Y Sleep debt elicits negative emotional reaction through diminished amygdala anterior cingulate functional connectivity. PloS One (2013) 8:e56578. 10.1371/journal.pone.0056578 23418586 PMC3572063

[71] Yoo SS Gujar J Hu P Jolesz FA Walker MP The human emotional brain without sleep — a prefrontal amygdala disconnect. Curr Biol (2017) 17:R877–R878. 10.1016/j.cub.2007.08.007 17956744

[72] Fox AS Shackman AJ The central extended amygdala in fear and anxiety: closing the gap between mechanistic and neuroimaging research. Neurosci Lett (2019) 693:58–67. 10.1016/j.neulet.2017.11.056 29195911 PMC5976525

[73] Shackman AJ Kaplan CM Stockbridge MD Tillman RM Tromp DPM Fox AS The neurobiology of dispositional negativity and attentional biases to threat: implications for understanding anxiety disorders in adults and youth. J Exp Psychopathol (2016) 7:311–42. 10.5127/jep.054015 PMC5130287 27917284

[74] Dugre JR Bitar N Dumais A Potvin S Limbic hyperactivity in response to emotionally neutral stimuli in schizophrenia: a neuroimaging meta-analysis of the hypervigilant mind. Am J Psychiatry (2019) 176:1021–9. 10.1176/appi.ajp.2019.19030247 31509006

[75] Simon EB Oren N Sharon H Kirschner A Goldway N Okon-Singer H Losing neutrality: the neural basis of impaired emotional control without sleep. J Neurosci (2015) 35:13194–205. 10.1523/JNEUROSCI.1314-15.2015 PMC6605430 26400948

